# Computing the Fatigue Life of Cold Spray Repairs to Simulated Corrosion Damage

**DOI:** 10.3390/ma14164451

**Published:** 2021-08-09

**Authors:** Daren Peng, Caixian Tang, Neil Matthews, Rhys Jones, Sudip Kundu, R. K. Singh Raman, Alankar Alankar

**Affiliations:** 1 Centre of Expertise in Structural Mechanics, Department of Mechanical and Aeronautical Engineering, Monash University, Wellington Rd, Clayton, VIC 3800, Australia; daren.peng@monash.edu (D.P.); sudip.kundu@monash.edu (S.K.); raman.singh@monash.edu (R.K.S.R.); 2 RUAG Australia, 836 Mountain Highway, Bayswater, VIC 3153, Australia; caixian.tang@ruag.com (C.T.); neil.matthews@ruag.com (N.M.); 3 ARC Industrial Transformation Training Centre on Surface Engineering for Advanced Materials, Faculty of Science, Engineering and Technology, Swinburne University of Technology, John Street, Hawthorn, VIC 3122, Australia; 4 Department of Mechanical Engineering, Indian Institute of Technology Bombay, Powai, Mumbai 400076, Maharashtra, India; alankar.alankar@iitb.ac.in

**Keywords:** cold spray repair, skin corrosion, durability, MIL-STD-1530D, EZ-19-01

## Abstract

This paper summarises the findings of an investigation into the durability of cold spray repairs, also known as supersonic particle deposition or SPD repairs, to simulated corrosion damage in AA7075-T7351 aluminium alloy specimens. A feature of this paper is that it is the first to show how to perform the mandatory durability analysis of repaired corroded structures, where the corroded material is first removed by machining and then repaired using cold spray, in a fashion consistent with the requirements delineated in USAF Structures Bulletin EZ-19-01, MIL-STD-1530D, and the US Joint Services Structural Guidelines JSSG2006.

## 1. Introduction

Cold spray, which is also termed supersonic particle deposition (SPD), is being increasingly used to repair military aircraft [1,2,3,4,5,6,7,8,9]. However, as explained in [1,2,3,5], to date the majority of applications are limited to non-structural applications. Exceptions to this are the eleven cold spray doublers applied to an F/A-18 full scale fatigue test [1], and the studies into the ability of cold spray to restore the (compressive) load bearing capacity of corroded P3C Orion upper wing skin structures [1,8]. In the case of the doublers applied to the F/A-18 [1] it was found that despite the high stresses, in one case a cold spray doubler was found to experience peak stresses in excess of 250 MPa, and despite the fact that the spectra were measured on an operational combat aircraft, which is a very aggressive spectrum, there was no failure or debonding of the doubler. Indeed, the doublers lasted (intact) for in excess of three design lifetimes, and failure in the fatigue test article occurred well away from any of the cold spray doublers. This coupled with the results associated with the extensive coupon test programs reported in [1,6], whereby cold spray repairs were subjected to fatigue loads representative of those seen by operational aircraft, suggests that cold spray can be used to ensure the structural integrity of load bearing aircraft structures. However, as outlined in USAF Structures Bulletin EZ-19-01 [10], MIL-STD-1530D [11], and the United States Joint Services Structural Guidelines JSSG2006 [12], the airworthiness certification of a cold spray repair to load bearing structure requires a durability life analysis (the term durability is defined in JSSG2006 to be: “Durability is the attribute of an airframe that permits it to resist cracking for a prescribed period of time.”) To this end, the present paper illustrates how to perform this analysis using a “building block” approach that is consistent with JSSG2006.

Whereas [13,14,15,16,17,18,19,20] revealed that, provided the deposition process is reasonably well optimised, cold spray coatings are exceptionally damage tolerant and that failure generally results from the nucleation, and subsequent growth, of cracking at the intersection between the cold spray coating and the substrate being repaired, there are currently no papers that explain how to perform the durability analysis needed to certify a cold spray repair to a load bearing (structural) member. In this context it should be noted that [13] extended the findings presented in [1], whereby the F/A-18 full scale test highlighted that the cold spray repairs were particularly durable and damage tolerant, to reveal that the damage tolerance of a 7075 powder cold spray coating on a 7075-T7351 substrate is such that even when the coating is as a notched failure, this was due to the nucleation and subsequent growth of cracks in the 7075-T7351 substrate, and that delamination and cracking of the coating did not occur until close to final failure of the specimens. However, despite the experimental evidence on the excellent durability and damage tolerance of cold spray doublers, there are no papers that explain how to perform the durability analysis needed for this failure mechanism.

The need for such durability analyses is highlighted in MIL-STD-1530D, which states that analysis is the key to certification, and that the role of testing is merely to validate or correct the analysis. However, other than [6] there very few papers which present a durability analysis of a cold spray repair in which the initial crack length is of the order of the equivalent initial damage size (EIDS) mandated [10,11,12], and where the predicted crack growth histories are compared with experimental measurements. Even then, in the study reported in [6] the nucleating crack was associated with corrosion pitting down a fastener hole that contained intergranular corrosion, and as such did not initiate either in the cold spray repair or at the intersection between the cold spray and the substrate being repaired. Consequently, the purpose of this paper is to illustrate how to perform the necessary damage tolerance and durability analysis in a fashion that is consistent with USAF Structures Bulletin EZ-19-01, MIL-STD-1530D, and Joint Services Structural Guidelines JSSG2006.

To this end this paper presents the results of preliminary testing and crack growth analysis of cold spray repairs to simulated corrosion where the damage has nucleated and subsequently grown from material discontinuities at the intersection between the cold spray coating and the substrate, and where the computed crack growth histories are compared with experimental measurements.

## 2. Materials and Methods

The durability analysis performed in this paper uses the Hartman–Schijve crack growth equation [21]. This equation takes the form:

da/dN = *D* (∆*κ*)*^n^* (1)

where ∆*κ* is the Schwalbe crack tip similitude parameter [22].

∆*κ* = (∆*K* − ∆*K_thr_*)/√(1 − *K*_max_/*A*) (2)

Here, *D* and *n* are material constants; *K* = *K*_max_ − *K*_min_, where *K*_max_ and *K*_min_ are the maximum and minimum values of *K* in a cycle; *K_thr_* is the fatigue threshold; and *A* is the apparent cyclic fracture toughness. This particular crack growth equation was used as it has been shown [6,21,23,24,25,26,27,28,29,30,31,32,33,34,35,36,37,38,39,40,41,42] to be able to compute the growth of small and long cracks in a range of both conventionally and additively manufactured materials, and hence is considered to be a valid crack similitude parameter. In this context it should be noted that [23] highlighted that ∆*κ* is a valid similitude parameter for cracking in structural adhesive. It should also be noted that, as also discussed in [23], a valid similitude parameter is essential both for analysis and for relating laboratory tests to operational aircraft.

An attractive feature of the formulation is that it is now known that, for a given material, the effect of different microstructures on crack growth reduces as the crack length reduces [36,40,41]. This effect is captured by allowing for changes in the terms *K_thr_* as the crack length increases, with the values of *D*, *A* and *n* being unchanged [21,36]. In this context, it has recently been shown [42] that conventionally manufactured, additively manufactured, and cold spray additively manufactured (CSAM) 316L stainless steels can all be represented by Equation (1) with the same values of *D* and *n*. Reference [42] also reported that for both conventionally manufactured and CSAM built 316L steel the term ∆*κ* was directly related to the change in the potential energy per cycle.

A detailed discussion of the computational fracture mechanics analysis methodology used to determine the stress intensity factor (*K*) from a knowledge of the stress field in the uncracked specimens is given in reference [23].

The data analysed in the present paper were taken from the experimental test program outlined in Section 3. These tests were performed using a 100 kN Instron (Norward, MA, USA) servo-hydraulic test machine using a sinusoidal wave form with a test frequency of 5 hz.

The references are either from publicly available peer reviewed journals, publicly available refereed conferences or texts. For references associated with conferences, books or book chapters the ISBN numbers are given. For those references found via Google searches, the web addresses are given. Thirty-six of the references are in journals that are listed in SCOPUS, and four can be found on US Department of Defense DTIC/US government websites. The book chapters and books referenced are all SCOPUS listed. Two references are available on the Det Norske Veritas (DNV) website. One reference is on the NLR (Amsterdam, The Netherlands) website. The keywords used to identify the references were: cold spray, supersonic particle deposition, durability, damage tolerance, and Hartman–Schijve.

## 3. Geometric Dimensions and Applied Marker Block Load Spectrum

Repairs to corrosion damage in operational aircraft frequently involve the damaged material being mechanically removed (machining/blending), leaving no remaining corrosion damage. Subsequent to the damage removal, repair normally entails one of the following: surface protection of the machined/blended area, filling of the machined/blended area (e.g., metal filled epoxies) or external patching. To simulate an embedded cold spray repair to corrosion damage, the test specimens were first machined and then repaired using “cold spray”.

**Figure 1:**
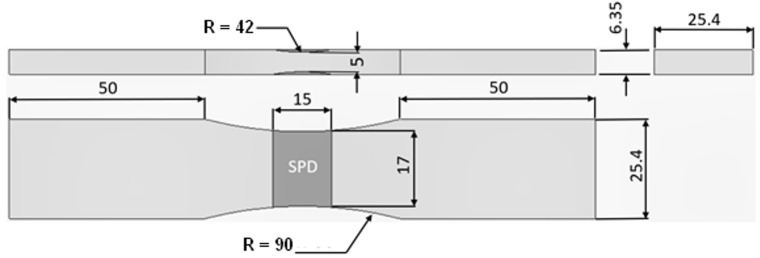
Dimensions of the “dog bone” specimen geometry.

The dimensions and geometry of the specimens used in this study are shown in Figure 1. The specimens were manufactured from AA7075-T351 aluminium alloy. A full width groove representing corrosion damage removal was machined into the specimens on both upper and lower faces. The depth of the groove was 0.675 mm, which represents a removal (loss) of approximately 20% of substrate material. These specimens were then “repaired” using cold spray 7075 power (Valimet AA7075, 53/±15 µm). The cold sprayed coating had compact structure with no visible pores.

The specimens tested were labelled 1, 2 and 3. The simulated corrosion cut (rework) was “repaired” using cold spray, and the repaired specimens were subjected repeatedly to the block load spectra outlined in Table 1 and Table 2. The thicknesses of the cold spray coatings associated with each specimen are given in Table 2. The cold spray repair (coating/deposition) was performed using a Plasma Giken (Saitama, Japan) Kinetik-8000 system using nitrogen, heated to between 500 to 800 °C, as the processing gas, with the 7075 powders accelerated at velocities from 500 to 800 m/s. Details about this technology are given in [2,3]. The precise details of the application process are proprietary to RUAG.

In this analysis the Young’s modulus and Poisson’s ratio of AA7075-T7351 were taken to be 73,000 MPa and 0.3, respectively. The Young’s modulus and Poisson’s ratio of cold spray coatings were taken to be 69,000 MPa and 0.3, respectively. These values were provided by RUAG and are from RUAG propriety data.

As per the requirement, delineated in JSSG206, to follow a “building block approach” the values of *D*, *A*, and *n* in Equation (1) were taken from prior studies [25]; namely, *D* = 1.86 × 10^−9^, *A* = 111 MPa √m, and *n* = 2. As shown in [6,21,25,26,39,40,41,42] the variability in the crack growth histories associated with durability analyses can be captured by allowing for variability in the local fatigue threshold (Δ*K_thr_*). The initial crack sizes (mm) and values of Δ*K_thr_* (MPa √m) used in the crack growth analyses are given in Table 3, where we see that these sizes, which were taken from fractographic measurements, were significantly smaller than that the mandated minimum EIDS given in [10,11,12].

**Figure 2:**
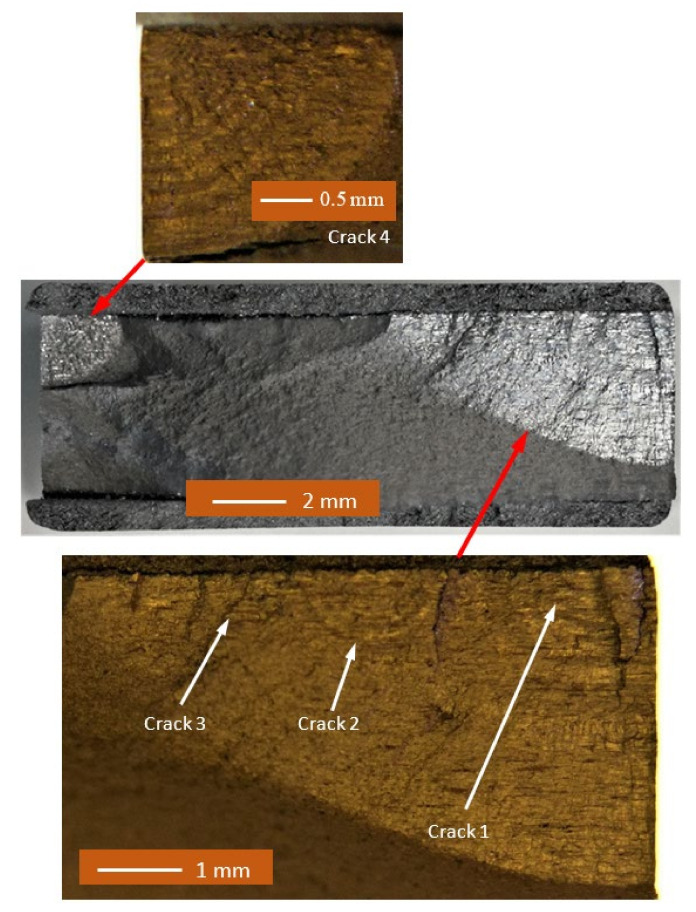
Failure surface associated with test specimen 1.

**Figure 3:**
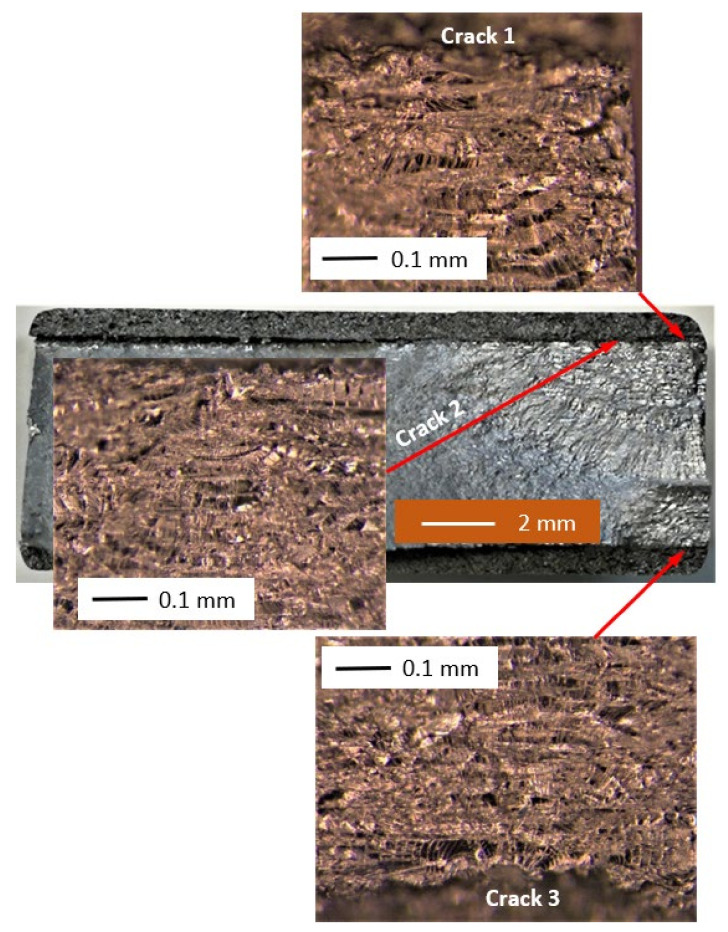
Failure surface associated with test specimen 2.

**Figure 4:**
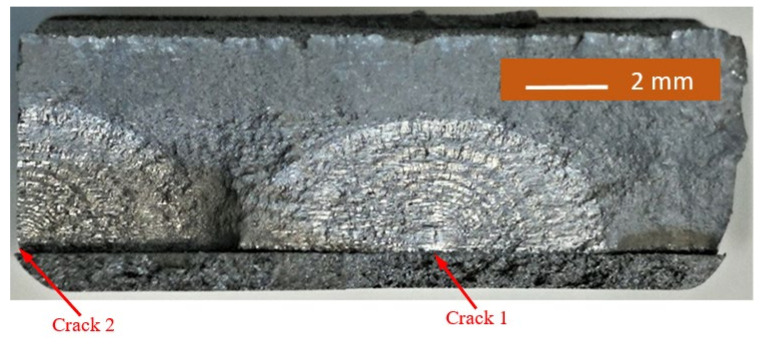
Failure surface associated with test specimen 3.

**Figure 5:**
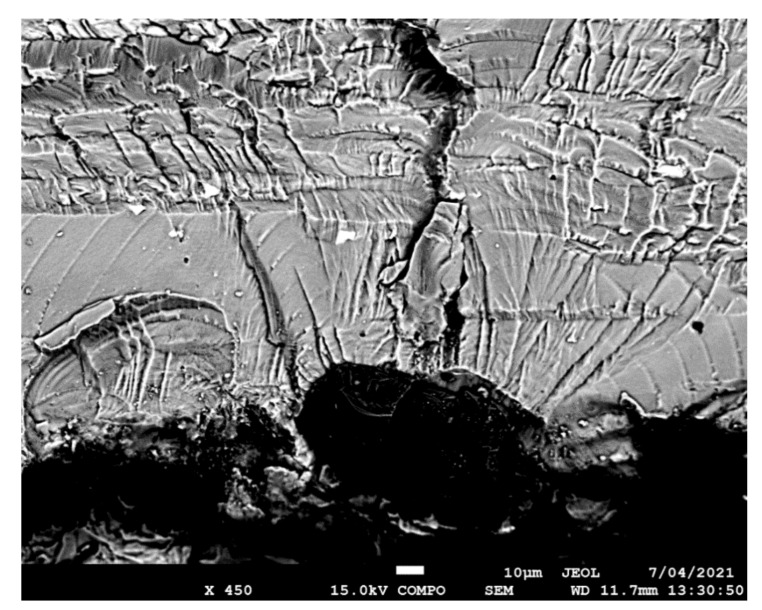
SEM of the lead crack (Crack 1) in specimen 1.

**Figure 6:**
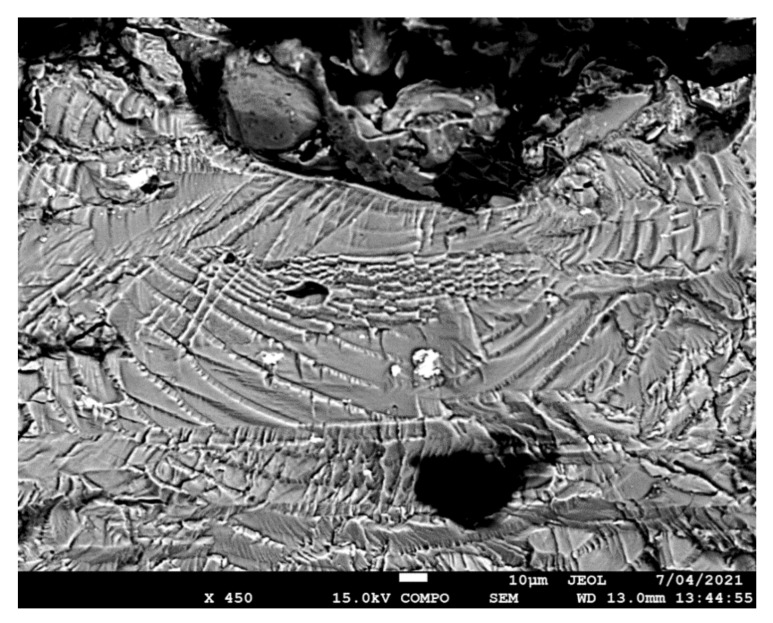
SEM of the lead crack (Crack 1) in specimen 2.

**Figure 7:**
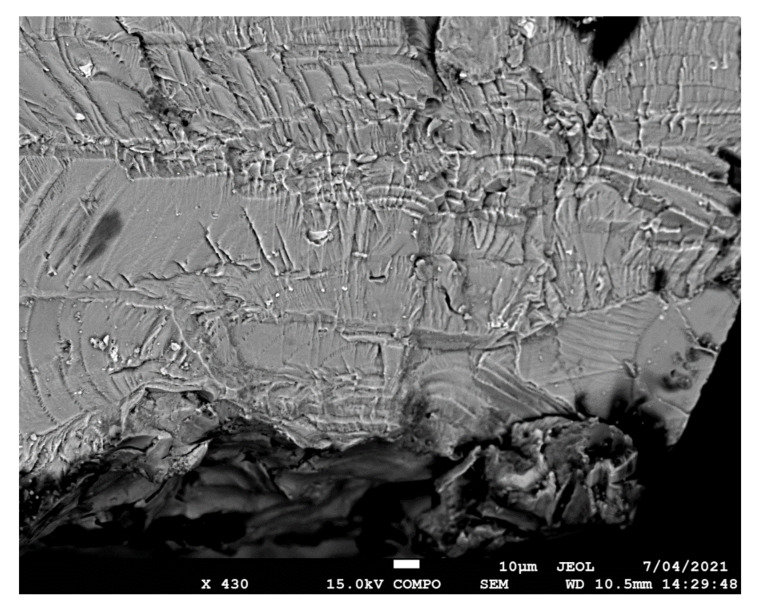
SEM of the lead crack (Crack 2) in specimen 3.

The failure surfaces associated with specimens 1–3 are shown in Figure 2, Figure 3 and Figure 4. These figures reveal that failure resulted for the nucleation of cracks in the AA7075-T7351 substrate and their subsequent growth into the AA7075-T7351. In all cases it was found that the cold spray coatings did not crack until late in the life of the specimen. SEM pictures of the fastest growing, i.e., lead, cracks are shown in Figure 5, Figure 6 and Figure 7, where a key feature is the relatively small size of the nucleating features. The crack identifiers associated with each of the cracks are shown in Figure 5, Figure 6 and Figure 7 and in Table 3. Figure 5, Figure 6 and Figure 7 also reveal that the sizes of the nucleating cracks are significantly smaller than the mandated minimum equivalent initial damage size (EIDS) given in [10,11,12] of 0.254 mm (0.01 inch).

**Figure 8:**
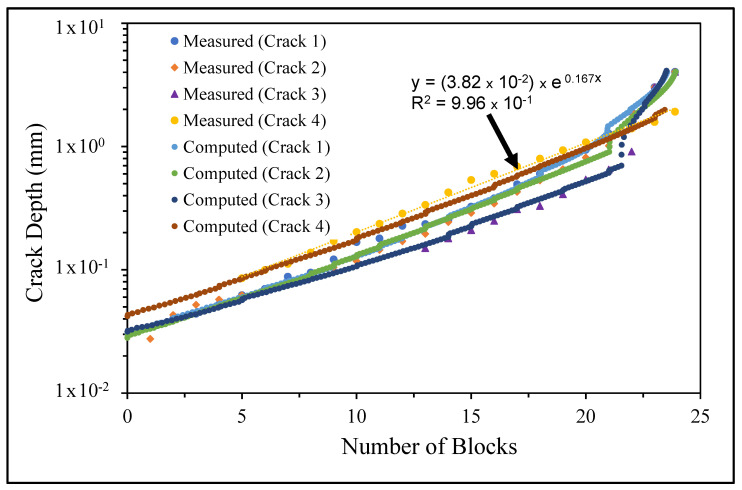
Measured and computed crack growth histories for specimen 1.

**Figure 9:**
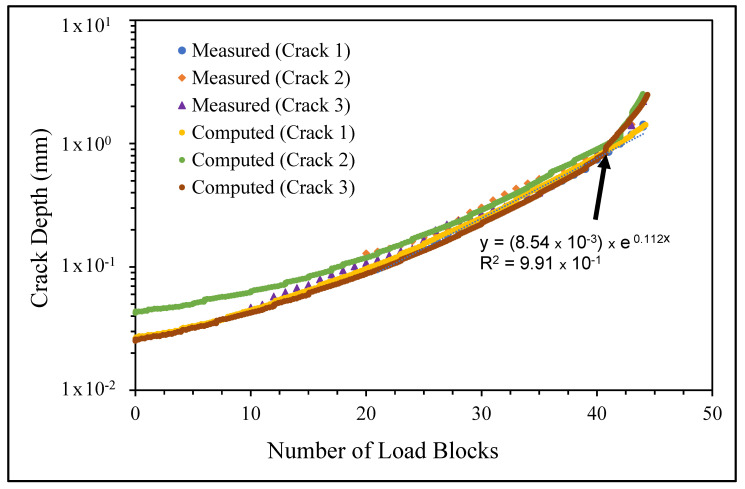
Measured and computed crack growth histories for specimen 2.

**Figure 10:**
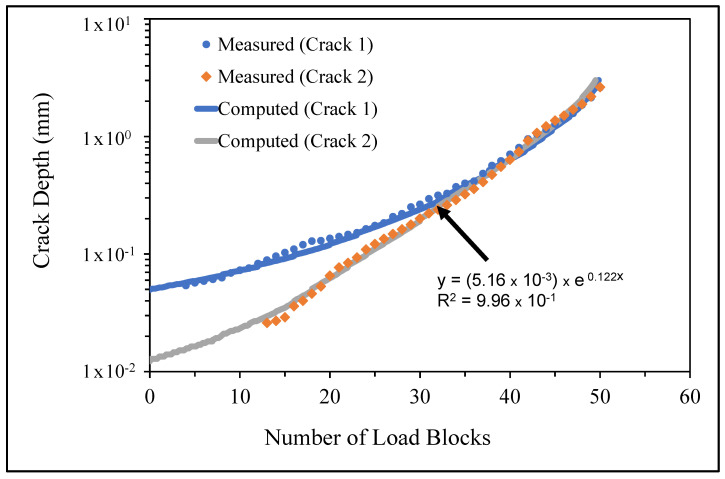
Measured and computed crack growth histories for specimen 3.

The measured and computed crack growth histories are shown in Figure 8, Figure 9 and Figure 10, where we see excellent agreement between the computed and measured crack growth histories. Table 3 reveals that the value of Δ*K_thr_* associated with these nine cracks falls within the range 0.0 to 0.3 that is commonly seen for small cracks in conventionally manufactured aluminium alloys [1,21,25,26,27,33,34,36,38,39]. In contrast, the Δ*K_thr_* range seen in [37] for the growth of long cracks in AA7075-T7351 aluminium alloy specimens tested under a FALSTAFF flight load spectrum was between (approximately) 1.1 and 1.79 MPa √m. This difference is typical of that seen by long and small cracks [21,25].

Figure 8, Figure 9 and Figure 10 also reveal that the growth of the fastest cracks (“lead cracks”) in this study is approximately exponential, and as such is consistent with that seen in both RAAF and USAF aircraft [43,44]. As a result, it follows from [45] that Miner’s rule may be applicable for estimating the life of cold spray repairs under variable amplitude loads.

## 4. Comparison of the Value of Δ*K_thr_* with Values Associated with Surface Breaking Cracks

It has recently been shown [6] that for the twenty cracks that nucleated and subsequently grew from the small sub-mm surface pits studied in [6] that had depths similar to the mandated [10,11,12] minimum equivalent initial damage size (EIDS) of 0.254 mm, the fatigue threshold Δ*K_thr_* lay in the range 0.35 to 1.2 MPa √m. However, as subsequently discussed in [39] a group of twenty cracks represents a limited data set, and as such may not necessarily capture the extent of the true variability in Δ*K_thr_*. To account for such limited data sets, [46,47,48,49] suggest the use of a statistical approach whereby the ‘A basis’ and ‘B basis’ properties are determined. An ‘A basis’ value equals the mean value minus three standard deviations and is the value above which at least 99% of the population of values is expected to fall with a confidence of 95% [46]. The ‘B basis’ value equals the mean value minus two standard deviations and is the value above which at least 95% of the population of values is expected to fall with a confidence of 95% [47]. The US Joint Services Structural Guidelines [12] recommend using A basis allowables. Det Norske Veritas (DNV) [48,49] recommends using the B basis allowables for fatigue design.

Jones et al. [39] subsequently reported that the mean value of Δ*K_thr_* associated with the twenty cracks studied in [6] was approximately 0.80 MPa √m, and that the standard deviation (σ) was approximately 0.24 MPa √m. This gives a mean minus two standard deviations (B basis) value of Δ*K_thr_* for cracks that nucleate and grow from surface breaking pits of 0.08 MPa √m. This value of Δ*K_thr_* is similar to the values given in Table 3 for the nine sub-surface interface cracks analysed in the present study.

## 5. Conclusions

MIL-STD-1530D notes that analysis is the key to certification, and that the role of testing is merely to validate or correct the analysis. USAF Structures Bulletins EZ-19-01 and MIL-STD-1530D mandate that a durability analysis must assume an EIDS of no greater than 0.254 mm; this value is taken from JSSG2006. This paper is unique in that it is the first to illustrate how to accurately compute the crack growth histories associated with naturally occurring cracks that nucleate and subsequently grow from material discontinuities associated with cold spray repairs to simulated corrosion damage, where the size of the nucleating cracks is significantly smaller than the equivalent initial damage size (EIDS) mandated in the JSSG2006, MIL-STD-1530D, and USAF Structures Bulletin EZ-19-01. As such, it is the first paper to illustrate how to perform the durability analysis mandated in EZ-19-01, MIL-STD-1530D and JSSG2006.

It is also shown that the growth of the fastest cracks (“lead cracks”) in this study is approximately exponential, and as such is consistent with that seen in both RAAF and USAF aircraft. As a result, Miner’s rule may be applicable for estimating the life of cold spray repairs under variable amplitude loads.

## Figures and Tables

**Table 1:** Block loading spectrum.

Spectrum	P_max_	Cycles	
	kN	R = 0.1	R = 0.8
3	30	300	15,000
5	26.77	300	15,000

**Table 2:** The block loading spectrum used in the fatigue tests and the cold spray thicknesses.

Specimen ID	Block Load Spectrum	Thickness * of Cold Spray (mm)
1	3	0.735
2	5	0.730
3	5	0.840

* This does not include the thickness of the cold spray used to fill the simulated corrosion cut.

**Table 3:** The initial crack lengths (EIDS) and values of Δ*K_thr_* (MPa √m) used in the crack growth analyses.

Specimen ID	Crack ID	Initial Crack Sizes Used in the Analysis (mm)	Δ*K_thr_* (MPa √m)
1	1	0.0300	0.06
	2	0.0275	0.07
	3	0.0310	0.05
	4	0.0410	0.07
2	1	0.0263	0.150
	2	0.0342	0.252
	3	0.0255	0.143
3	1	0.0480	0.2
	2	0.006	0.12

## Data Availability

This is an ongoing study and the data will be made available on the completion of the project.
